# Influence of Regular Exercise on Body Fat and Eating Patterns of Patients with Intermittent Claudication

**DOI:** 10.3390/ijms160511339

**Published:** 2015-05-18

**Authors:** Anthony Leicht, Robert Crowther, Jonathan Golledge

**Affiliations:** 1 College of Healthcare Sciences, James Cook University, Townsville, QLD 4811, Australia; 2 School of Health and Wellbeing, University of Southern Queensland, Springfield, QLD 4350, Australia; E-Mail: Robert.Crowther@usq.edu.au; 3 The Vascular Biology Unit, Queensland Research Centre for Peripheral Vascular Disease, College of Medicine and Dentistry, James Cook University, Townsville, QLD 4811, Australia; E-Mail: Jonathan.Golledge@jcu.edu.au; 4 Department of Vascular and Endovascular Surgery, the Townsville Hospital, Townsville, QLD 4814, Australia

**Keywords:** body fat, skinfold, walking, claudication, diet, training

## Abstract

This study examined the impact of regular supervised exercise on body fat, assessed via anthropometry, and eating patterns of peripheral arterial disease patients with intermittent claudication (IC). Body fat, eating patterns and walking ability were assessed in 11 healthy adults (Control) and age- and mass-matched IC patients undertaking usual care (*n* = 10; IC-Con) or supervised exercise (12-months; *n* = 10; IC-Ex). At entry, all groups exhibited similar body fat and eating patterns. Maximal walking ability was greatest for Control participants and similar for IC-Ex and IC-Con patients. Supervised exercise resulted in significantly greater improvements in maximal walking ability (IC-Ex 148%–170% *vs.* IC-Con 29%–52%) and smaller increases in body fat (IC-Ex −2.1%–1.4% *vs.* IC-Con 8.4%–10%). IC-Con patients exhibited significantly greater increases in body fat compared with Control at follow-up (8.4%–10% *vs.* −0.6%–1.4%). Eating patterns were similar for all groups at follow-up. The current study demonstrated that regular, supervised exercise significantly improved maximal walking ability and minimised increase in body fat amongst IC patients without changes in eating patterns. The study supports the use of supervised exercise to minimize cardiovascular risk amongst IC patients. Further studies are needed to examine the additional value of other lifestyle interventions such as diet modification.

## 1. Introduction

Peripheral arterial disease (PAD) is an occlusive vascular condition that affects up to 15% of the population [1,2,3] with patients typically exhibiting reduced peripheral blood flow, decreased walking ability and poor quality of life [4,5,6]. Initial treatment normally involves risk factor control including medications and lifestyle modification in line with international guidelines [7]. The key lifestyle changes include smoking cessation, a controlled diet and increased performance of regular exercise [7]. Such lifestyle modifications have been demonstrated to promote weight loss or weight maintenance in healthy adults [8] and are recommended for obese individuals [7]. Many studies have reported that regular supervised exercise improves walking ability for PAD patients [9,10,11]. Whether such exercise programs influence diet, eating patterns and body composition is less clear for this population. Studies examining the effect of exercise on energy intake and food selection have reported inconsistent results for animal and human studies [12].

Previous reports suggested that PAD patients did not consume a diet in line with current recommendations [13]. It was reported that PAD patients ate high amounts of saturated fat, cholesterol and sodium, and low levels of fibre and folate [14]. A large cross-sectional analysis reported that high intake of vitamins A, C and B6, fibre, folate and omega-3 fatty acids was associated with lower prevalence of PAD within the USA population [15]. Consumption of a Mediterranean diet was also associated with a lower prevalence of PAD [16]. In contrast, another study suggested there was no relationship between PAD prevalence and diet when corrected for energy intake and physical activity levels [17]. A recent review suggested that behavioural weight management programs incorporating both diet and physical activity were optimal to reduce weight in overweight or obese adults [18].

Only one previous study has examined the effect of a combined dietary and exercise intervention for PAD patients to our knowledge [19]. These authors evaluated a lifestyle program, including dietary recommendations, moderate exercise and a smoking cessation intervention, in 13 PAD patients over 15 months. The program resulted in a reduction in the number of patients smoking >1 packet per day (54.2% down to 7.7%), an increase in physical activity (number of patients walking >1 km from 13% up to 77%), and a reduction in caloric intake (10%), saturated fat (10%) and cholesterol (10%). The authors concluded that a lifestyle educational program focussing on diet, exercise and smoking cessation improved the risk factor profile of PAD patients. The program did not however have any effect on walking ability.

The aim of the current study was to examine the impact of a regular supervised exercise program on body fat and eating patterns of PAD patients with intermittent claudication (IC). It was hypothesised that regular exercise would significantly reduce body fat and alter eating patterns [20] in patients with IC.

## 2. Results and Discussion

Twenty patients with IC and 11 age- and mass-matched, healthy adults (Control) volunteered for this study and their characteristics are shown in Table 1. All groups were similar in age, height, mass, body mass index (BMI), girths, skinfolds (except mid-axilla) and body fat (%) at entry (Table 1). Walking ability was similar for each group of IC patients at entry and significantly lower than that for healthy controls (Table 1). Eating patterns were similar for all groups at entry except for weekly beer consumption. Weekly beer consumption was significantly greater for patients with IC undertaking usual care (IC-Con) compared with healthy controls (Q13a, Table 2).

**Table 1:** Entry characteristics including anthropometric measures, body fat and walking ability amongst healthy adults (Control) and aged-matched patients with intermittent claudication undertaking usual care (IC-Con) or regular supervised exercise training (IC-Ex).

Variable	Control (*n* = 11)	IC-Con (*n* = 10)	IC-Ex (*n* = 10)
Age (years)	68.1 ± 6.8	66.5 ± 6.9	71.3 ± 8.5
Height (cm)	167.8 ± 8.2	165.1 ± 9.0	165.7 ± 8.9
Mass (kg)	70.7 ± 8.4	74.3 ± 19.9	80.7 ± 15.3
BMI (kg/m^2^)	25.2 ± 3.2	27.0 ± 5.6	29.2 ± 4.1
Waist Girth (cm)	85.5 ± 9.3	92.1 ± 16.0	96.1 ± 11.5
Hip Girth (cm)	98.3 ± 7.3	101.3 ± 12.3	108.7 ± 9.5
Waist:Hip ratio	0.9 ± 0.1	0.9 ± 0.1	0.9 ± 0.1
**Skinfold (mm)**			
Triceps	18.3 ± 8.7	18.5 ± 6.6	23.3 ± 8.1
Subscapular	16.8 ± 8.7	22.0 ± 7.7	26.8 ± 12.3
Biceps	8.8 ± 5.7	9.8 ± 4.3	14.5 ± 9.0
Iliac Crest	16.4 ± 9.5	18.6 ± 10.6	29.5 ± 19.9
Supraspinale	13.4 ± 10.0	13.1 ± 9.0	28.4 ± 20.8
Abdominal	27.4 ± 12.4	29.6 ± 13.1	44.5 ± 19.1
Thigh	23.7 ± 11.1	22.6 ± 10.5	31.0 ± 17.1
Calf	16.4 ± 9.7	12.7 ± 5.8	20.2 ± 13.7
Mid-axilla	15.5 ± 7.2	18.3 ± 9.7	28.2 ± 12.8 *^,†^
**Body Fat (%)**			
LifeSize [21]	33.8 ± 19.3	26.5 ± 7.5	36.3 ± 18.9
Durnin & Womersley [22]	25.9 ± 10.6	29.2 ± 7.2	30.6 ± 8.3
Katch & McArdle [23]	25.8 ± 13.3	29.5 ± 13.7	25.9 ± 19.6
Sloan [24]	19.5 ± 8.8	24.6 ± 7.2	26.9 ± 13.1
Wilmore & Behnke [25]	24.4 ± 8.4	24.9 ± 7.3	26.3 ± 11.5
Thorland *et al.* [26]	22.9 ± 10.7	26.5 ± 10.1	29.1 ± 14.1
Withers *et al.* [27]	22.9 ± 10.4	23.0 ± 6.3	27.8 ± 10.7
**Walking Ability**			
Pain Free Walking Distance (m)	No pain at all	103 ± 88	115 ± 48
Maximal Walking Distance (m)	977 ± 402	251 ± 163 *	285 ± 135 *

Values are mean ± SD; BMI—Body mass index; * *p* < 0.05 *vs.* Control ^†^ *p* < 0.05 *vs.* IC-Con.

**Table 2:** Eating patterns amongst healthy adults (Control) and aged-matched patients with intermittent claudication undertaking usual care (IC-Con) or supervised exercise (IC-Ex) prior to and at 3- and 12-months of the study.

Group	0-Month	3-Months	12-Months
**Q1**			
Control	4 (3, 4.5)	4 (3.5, 4.5)	4 (2.5, 4)
IC-Con	4 (3, 4)	4 (3, 4)	4 (3.25, 4)
IC-Ex	4 (3, 4.75)	4 (3.25, 5)	4 (3.25, 4)
**Q2**			
Control	3 (2.5, 4)	4 (2.5, 4)	3 (2, 4)
IC-Con	4 (3.25, 4.75)	4 (3.25, 4)	4 (4, 4)
IC-Ex	4 (4, 5)	4 (3.25, 4)	4 (4, 4)
**Q3**			
Control	4 (3, 4.5)	4 (4, 4.5)	4 (3.5, 4)
IC-Con	4 (3.25, 5)	4 (3.25, 5)	4 (3.25, 5)
IC-Ex	4 (2.25, 4)	3 (2, 4)	4 (3.25, 4)
**Q4**			
Control	4 (4, 4.5)	4 (4, 4)	4 (4, 4)
IC-Con	4 (4, 4)	4 (4, 4)	5 (4, 5)
IC-Ex	4 (4, 5)	4 (4, 4)	4 (4, 4.75)
**Q5**			
Control	5 (3.5, 5)	4 (4, 5)	5 (4, 5)
IC-Con	3 (2.25, 4)	3 (3, 4)	3 (2, 3) *
IC-Ex	4 (2.5, 4.75)	4 (3, 5)	4 (3, 4) *^,†^
**Q6**			
Control	3 (3, 3)	3 (2, 3)	3 (2, 3)
IC-Con	2 (2, 2.75)	2 (2, 3)	2 (2, 3)
IC-Ex	2 (2, 2.75)	2.5 (2, 3)	2 (2, 2.75)
**Q7**			
Control	3 (2, 3.5)	3 (2, 3)	3 (2, 4)
IC-Con	2 (2, 2)	2 (2, 2.75)	2.5 (2, 3)
IC-Ex	2 (2, 2.75)	3 (2, 3)	2 (2, 3.75)
**Q8**			
Control	4 (3, 4)	4 (3.5, 4)	4 (3.5, 4)
IC-Con	4 (3, 4)	3 (3, 4)	4 (3, 4)
IC-Ex	3.5 (3, 4)	3 (3, 4)	3.5 (3, 4)
**Q9**			
Control	2 (2, 2)	2 (2, 2)	2 (2, 2)
IC-Con	2 (2, 2)	2 (2, 2)	2 (2, 2)
IC-Ex	2 (2, 2)	2 (2, 2)	2 (2, 2)
**Q10**			
Control	3 (2.5, 3)	3 (2.5, 3)	3 (3, 3)
IC-Con	3 (2, 3)	3 (2.25, 3.75)	3 (2, 3.75)
IC-Ex	2.5 (2, 3)	3 (2, 3)	2.5 (2, 3.75)
**Q11**			
Control	2 (1, 3.5)	1 (1, 4.5)	1 (1, 3.5)
IC-Con	2 (1, 3.75)	1.5 (1, 4.75)	2.5 (1, 4.75)
IC-Ex	1 (1, 3.75)	1.8 (1, 4.75)	1 (1, 2)
**Q12**			
Control	1 (1, 1)	1 (1, 1)	1 (1, 1)
IC-Con	1 (1, 1)	1 (1, 1.75)	1 (1, 1)
IC-Ex	1 (1, 1)	1 (1, 1.5)	1 (1, 1)
**Q13a**			
Control	1 (1, 1)	1 (1, 1)	1 (1, 1)
IC-Con	1 (1, 2) *	1 (1, 2)	1 (1, 1)
IC-Ex	1 (1, 1)	1 (1, 1.3)	1 (1, 1)
**Q13b**			
Control	1 (1, 1.5)	1 (1, 2)	1 (1, 2)
IC-Con	1 (1, 1)	1 (1, 1)	1 (1, 1)
IC-Ex	1 (1, 1)	1 (1, 1)	1 (1, 1)
**Q13c**			
Control	1 (1, 1)	1 (1, 1)	1 (1, 1)
IC-Con	1 (1, 1)	1 (1, 1)	1 (1, 1)
IC-Ex	1 (1, 1)	1 (1, 1)	1 (1, 1)

Values are median (interquartile range) responses to eating patterns questionnaire (see Appendix A); * *p* < 0.05 *vs.* Control ^†^ *p* < 0.05 *vs.* IC-Con.

### 2.1. Impact of Regular Supervised Exercise

Regular supervised exercise resulted in significantly greater improvements in walking ability for patients with IC (IC-Ex) compared to both control groups (IC-Con and Control) at 3- and 12-months (Table 3). Improvements in walking ability were similar for Control and IC-Con at 3- and 12-months (Table 3).

#### 2.1.1. Body Fat

Significantly greater reductions in body mass, and supraspinale and mid-axilla skinfolds were noted for patients undertaking supervised exercise (IC-Ex) compared to the usual care (IC-Con) group (Table 3). The reductions in skinfolds for IC-Ex patients remained at 12-months (Table 3). Similarly, increases in body fat (%) were smaller for the IC-Ex compared to the IC-Con group at 3- (via LifeSize equation) and 12-months (via LifeSize and Withers *et al.* equations) and comparable to that of healthy controls (Table 3). In contrast, IC-Con patients exhibited significantly greater increases in body fat (%) compared with the Control adults at 3- (via LifeSize equation) and 12-months (via LifeSize and Withers *et al.* equations, Table 3).

#### 2.1.2. Eating Patterns

Eating patterns were similar for all groups at 3- and 12-months of the study (Table 2).

**Table 3:** Absolute or relative (%) change in walking ability, anthropometric measures and body fat of healthy adults (Control) and age- matched patients with intermittent claudication undertaking 3 and 12 months of usual care (IC-Con) or regular supervised exercise training (IC-Ex).

Variable	Time Point	Control (*n* = 11)	IC-Con (*n* = 10)	IC-Ex (*n* = 10)
**Walking Ability (%)**				
Pain free walking distance	3 months	N/A	47.6 ± 66.4	166.4 ± 143.4 ^#^
	12 months	N/A	102.2 ± 160.8	242.1 ± 166.8 ^†^
Maximal walking distance	3 months	30.5 ± 62.8	29.1 ± 67.0	148.1 ± 154.6 *^,†^
	12 months	18.9 ± 47.9	52.3 ± 127.6	169.7 ± 166.0 *^,†^
Body mass (%)	3 months	−1.1 ± 2.6	0.7 ± 2.5 *	−1.4 ± 2.0 ^†^
	12 months	−2.4 ± 4.1	−0.2 ± 2.9	0.9 ± 3.6
**Skinfold (%)**				
Supraspinale	3 months	2.1 ± 17.9	3.7 ± 10.3	−19.7 ± 21.4 *^,†^
	12 months	2.9 ± 27.0	16.6 ± 40.3	−26.0 ± 28.7 *^,†^
Mid-axilla	3 months	−7.0 ± 25.3	6.1 ± 15.4	−17.3 ± 23.5 ^†^
	12 months	3.9 ± 32.2	−3.2 ± 19.0	−23.6 ± 17.7 *^,†^
**Body Fat (Absolute)**				
LifeSize [21]	3 months	−0.6 ± 1.9	10.0 ± 10.8 *	1.4 ± 8.8 ^#^
	12 months	−1.4 ± 2.1	8.4 ± 11.1 *	−2.1 ± 9.9 ^#^
Withers *et al.* [27]	3 months	−0.6 ± 2.0	1.8 ± 3.3	−1.3 ± 4.1
	12 months	−1.2 ± 1.8	0.8 ± 3.5 *	−3.7 ± 5.4 ^†^

Values are mean ± SD; N/A not applicable; ^#^ *p* < 0.06 *vs.* IC-Con; ^†^ *p* < 0.05 *vs.* IC-Con; * *p* < 0.05 *vs.* Control.

The current study demonstrated that regular, supervised exercise significantly increased maximal walking ability for IC patients as previously reported [11,28,29,30]. The main novel finding we reported was that supervised exercise was associated with less increase in body fat compared to usual care. Previous studies have documented significant improvements in walking ability with both short- [31] and long-term [10,32,33] training however, changes in body fat following exercise training have been rarely reported for PAD patients. We previously reported a strong association between obesity and the severity of IC and complications in PAD patients [34]. Others have also associated obesity with poorer walking ability [35], limited exercise performance [36] and reduced improvements in walking following supervised exercise [37] amongst PAD patients. In contrast to those previous reports, we recently reported that obesity was associated with increased survival amongst a group of approximately 1500 patients with heterogeneous presentations of peripheral vascular disease [38]. This obesity paradox has been previously reported in patients with a range of significant medical conditions [39,40,41]. While these above reports appear contradictory, there may be some reasonable explanations. It is possible that amongst patients with milder types of PAD, such as those with IC suitable for exercise training, obesity is associated with a poorer prognosis. In patients with more severe types of arterial disease, such as those with critical limb ischemia, obesity may have different prognostic significance. For these patients, obesity may be a marker of less advanced disease, reduced frailty and greater robustness to withstand multiple hospital admission and events typically experienced by these individuals.

In the current study, we showed that supervised walking training resulted in reduced body fat accumulation for IC patients. The smaller body fat increase was independent of physical activity levels, additional to the supervised exercise program, that were unchanged during the 12 month study period [11]. Recently, Li and colleagues concluded in their review [20] that exercise training frequency, quantity and mode (aerobic exercise like walking) had an important influence on weight loss. The current results provide further support that aerobic exercise can assist with body fat modification. The reduced fat accumulation was primarily demonstrated within the torso. Several skinfold measurements at the supraspinale and mid-axilla regions were significantly reduced in patients randomized to supervised exercise. Skinfolds assessed at the iliac crest and abdomen were also reduced in patients receiving supervised exercise although the trend was not significantly different to the control group. Abdominal fat accumulation has been associated with poorer exercise performance [36] and increased complications [34,42]. Collectively, these results suggest that regular supervised exercise results in less accumulation of subcutaneous body fat which may contribute to better patient outcomes.

A novel finding of the current study was the increase in body fat (via LifeSize and Withers *et al.* equations) over 3–12 months for patients undertaking usual care (IC-Con). Further, this change occurred with similar eating patterns to that of other IC patients and healthy, aged-matched adults. To our knowledge, no study of IC patients has reported this detrimental change in patient’s body fat and provides further support for the inclusion of exercise within the care plans for IC [7]. The previously reported associations between obesity and the severity, functional impairment and complications of PAD [34,35,36,37] highlight the importance of interventions to limit body fat in IC patients. The current results indicate the importance of supervised exercise for IC patients which is not routinely available.

The body fat change for patients receiving supervised exercise occurred independently of eating patterns. Leone and colleagues [43] reported that training responses for PAD patients were similar regardless of the presence of risk factors such as smoking, hypertension, and obesity. The current results extend these findings to demonstrate the negligible impact of eating patterns on body fat changes during exercise interventions. Several epidemiological studies have reported greater PAD prevalence amongst individuals that consume greater amounts of saturated fat and reduced intake of some vitamins [44]. Very few of these analyses however have adjusted for exercise level amongst patients and therefore, it is not currently clear whether physical activity or exercise, nutrition or both are important in determining the prevalence and complications of PAD.

Supervised exercise has demonstrated positive gains for patients with IC however whether this intervention should be combined with a nutritional program is not currently clear. Such lifestyle programs have reported benefits amongst a number of different populations such as healthy adults [18] and patients with metabolic syndrome [45]. Similar programs may also result in health benefits for PAD patients. Ramirez-Tortosa and colleagues [19] reported that PAD patients who undertook a combined diet and moderate exercise intervention over 15 months exhibited greater physical activity levels and better diet. Future studies are needed to identify the optimal design of exercise focused interventions and the advantage of combining these with a nutritional program.

The current study has a number of limitations. Analysis was limited to a small sample of PAD patients as has been the case in most previous studies of supervised exercise [19]. Eating patterns were determined from a questionnaire alone rather than more detailed assessments including total caloric intake from food diaries or direct observations [15]. Finally, body fat was assessed from skinfold measurements that required significant technical expertise and use of a number of body fat% equations of variable results. For example, the body fat% change at 3-months for IC-Con using the LifeSize equation was substantially greater than that for the Withers *et al.* equation (Table 3). Information concerning the LifeSize equation was not available due to proprietary restrictions but the methodological differences between body fat% equations may influence interpretations. Future studies using more advanced techniques such as DEXA, which can assess both fat and lean muscle mass, may better clarify the specific body composition responses to supervised exercise for PAD patients.

## 3. Experimental Section

### 3.1. Participants

The design of this study has been previously reported [11,28,29,30]. Participants presenting with IC due to PAD were recruited based on the findings of history and physical examination following consultation with a vascular physician. Diagnosis of PAD was confirmed by ankle-brachial pressure (ABI) index <0.9 and lower limb artery stenoses or occlusions demonstrated on arterial duplex and/or computed tomography angiography. For comparison, a group of healthy, age- and mass-matched adults (Control, *n* = 11) that were free of PAD (ABI > 0.9) were recruited from the local community. All participants provided written informed consent to participate with the study approved by the Townsville Health Service District Institutional Ethics Committee (01/03, 16 January 2003) and James Cook University Human Research Ethics Committee (H1640, 28 August 2003).

### 3.2. Assessments

Participants arrived at the laboratory early in the morning after an overnight fast and completed a questionnaire inquiring about medical history, medications, comorbidities, smoking history [11] and eating patterns. Participants were asked about their eating of protein, dairy, desserts, fats, carbohydrates, fruit, vegetables, fast foods, salty foods, and alcohol consumption using a questionnaire (Wellsource Inc., Clackamas, OR, USA; Appendix A).

Participant’s height was measured via stadiometer (Seca 220, Seca Scales, Hamburg, Germany) and body mass was assessed using scales (TANITA TBF 521, TANITA Corp., Arlington Heights, IL, USA). Body mass index (BMI) was calculated based upon height and mass and expressed in kg∙m^−2^. Body fat was then determined from measurements using a restricted anthropometric profile as previously described [46,47]. The restricted anthropometric profile consisted of 9 skinfold measurements (*i.e.*, triceps, biceps, subscapular, supra-iliac, abdominal, supra-spinale, mid-axilla, thigh and calf) (Table 1). All skinfold measurements were assessed in duplicate using calibrated calipers in accordance with international anthropometry guidelines [46]. Body fat percentage was calculated using a specialized computer program (*i.e.*, LifeSize [21]; Human Kinetics, Champaign, IL, USA). Body fat (%) was also calculated using the previously employed equations of Durnin and Womersley [22], Katch and McArdle [23], Sloan [24], Wilmore and Behnke [25], Thorland *et al.* [26] and Withers *et al.* [27] within LifeSize [21,48]. Waist to hip ratio was assessed using a steel tape [46].

Exercise or walking capacity was assessed for each participant as previously described following familiarisation [11]. Briefly, participants completed an incremental exercise test on a motorized treadmill (Trackmaster TMX55, Full Vision, Newton, KS, USA). The walking protocol was conducted at a constant speed of 3.2 km·h^−1^ with an incline of 0% for the first 2 min that was increased by 2% every 2 min until volitional exhaustion [49] or a maximum of 25 min. Exercise capacity was assessed as walking distance that was pain free (PFWD) using a claudication pain scale [50] and maximal walking distance (MWD).

### 3.3. Randomisation and Follow-Up

Participants with PAD were randomly allocated using sealed envelopes to undertake supervised exercise (IC-Ex, *n* = 10) or usual care (IC-Con, *n* = 10) over 12 months. As previously reported [28], usual care consisted of standardised treatment in accordance with international guidelines [7]. Follow-up assessments were performed at 3- and 12-months. These time intervals were chosen since significant improvements in walking ability have been reported at 2–4 [31] and 12 months [10,11,32,33] previously.

### 3.4. Exercise Training and Testing

Patients randomized to receive supervised exercise completed three sessions of treadmill walking each week over 12 months at an intensity required to induce maximal claudication pain as previously described [11]. Initial walking sessions aimed to achieve 20 min of continuous walking with subsequent sessions increasing duration progressively to a maximum of 45 min of continuous walking. Workloads were monitored and amended as needed to ensure attainment of maximal claudication pain during each walking session. Compliance with this exercise program was good (>70%) over the 12-month period as previously described [29].

### 3.5. Statistical Analysis

Data are presented as mean ± SD or median and interquartile range after assessment of data normality and data type (nominal or categorical). As a result of the modest sample size and assessment of data normality, comparisons between groups at each time point (0-, 3- and 12-months) were conducted using Kruskal-Wallis and post-hoc Mann-Whitney U tests. Changes (e.g., 3- *vs.* 0-months) in body fat and walking capacity over time were also examined and expressed as absolute (e.g., body fat %) or relative differences (e.g., skinfold, walking distance). All comparisons were conducted using the Statistical Program for the Social Sciences (v20, IBM Corp., Armonk, NY, USA) and the level of significance was set at 0.05.

## 4. Conclusions

Regular, supervised exercise significantly improved maximal walking ability and minimised increases in body fat accumulation. Long-term supervised exercise programs have capacity to reduce cardiovascular risk amongst IC patients. Eating patterns were unchanged amongst IC patients involved in supervised exercise. Future studies are needed to examine the impact of combining dietary interventions with supervised exercise in PAD patients.

## Appendix A: Eating Pattern Survey (Wellsource Inc, Clackamas, OR, USA)

Mark the choices below that best describe your regular eating pattern.

1. Meat/Protein foods

Eat red meat (fat not trimmed), sausages, hamburgers or luncheon meats (devon, salamis) most days.	□ 1
Eat some red meat (not trimmed) or poultry with skin more than 4 times per week, and fish occasionally.	□ 2
Eat mostly lean red meats, some skinless poultry, and some fish but choose steaks or dishes such as lasagne often when eating out.	□ 3
Eat moderate amounts of very lean meats and mostly skinless poultry and fish several times a week.	□ 4
Eat only small portions of very lean red meat or skinless poultry, have fish often and some legumes.	□ 5

2. Dairy Products/Eggs. Indicate the Kinds of Dairy Products You Usually Eat.

**High fat** dairy products include butter, cream, sour cream (include light sour cream). Cheeses (include those marked fat-reduced but not those labelled 7% fat). Ice cream, full cream milk (except for small amounts added to tea or coffee), regular yoghurt (in amounts greater than 100 g).

**Low fat** dairy products include skim or low fat milk, low fat yoghurt, cottage cheese.

Nearly always eat the high fat products.	□ 1
Eat mostly the high fat products, some low fat.	□ 2
Eat both about the same.	□ 3
Eat mainly low fat products and only small quantities of high fat.	□ 4
Eat only low fat products.	□ 5

3. Desserts/Snacks. Indicate the Kind of Desserts/Snacks You Usually Eat.

**High fat desserts/snacks** include: ice-cream, cakes, pastries, pies or tarts, chocolate desserts, frozen yoghurt, mousse or cream caramel, home baked desserts which include butter or margarine, biscuits (sweet or savoury except low fat crispbread), chocolate bars of any kind, caramels.

**Low fat desserts/snacks** include: fruit (fresh, dried or canned), fruit salads, low fat yoghurt (regular or frozen), low fat crispbread (rye or oat based), bread, whole-wheat breakfast biscuits, jelly.

Eat at least one item from the high fat products most days.	□ 1
Eat at least one item from the high fat products at least every second day plus some low fat products.	□ 2
Eat both about the same.	□ 3
Eat mostly low fat products, high fat not more than once a week.	□ 4
Eat only low fat products or none at all.	□ 5

4. Cooking Fats/Food Preparation. Indicate the Way You Usually Prepare Foods or the Kinds of Cooked Foods You Choose When Eating Out.

**High fat methods**: Deep-frying in oil or fat. Shallow frying in butter, margarine, lard, dripping or oil. Add extra butter, margarine, cream or oil to foods (e.g., pasta, vegetables, sauces).

**Low fat methods**: Grilling, barbecuing, using only a little oil on a non-stick pan, roasting on a rack, microwaving without adding fat, making casseroles, and allowing the fat to settle on top before removing it. Using low fat dressings and sauces (e.g., based on tomato, wine, herbs, vegetables), substituting low fat yoghurt or evaporated skim milk for cream or butter.

Food nearly always prepared the high fat way.	□ 1
Food mostly prepared the high fat way.	□ 2
Food prepared both ways about the same.	□ 3
Food usually prepared the low fat way, do not have foods prepared the high fat way except for occasional restaurant meals.	□ 4
Food prepared only the low fat way.	□ 5

5. Breads, Grains and Cereals. Indicate the Kind of Breads and Grains You Usually Eat.

**High fibre** products include wholemeal or wholegrain or increase fibre breads, rolls, muffins or fruit loaves, wholegrain breakfast cereals (e.g., whole-wheat breakfast biscuits, rolled oats, muesli, bran, wheat or oat cereals), wholemeal pasta, brown rice, millet, buckwheat, corn or rye products.

**Low fibre** or refined products include white breads, cereals such as cornflakes or rice bubbles, white rice or pastas.

**1**	**2**	**3**	**4**	**5**
□	□	□	□	□
Always choose low fibre or refined products	Eat mostly low fibre or refined products	Eat both about the same	Eat mainly high fibre products	Always choose high fibre products

6. Fruit. How Many Servings of **High Fibre Fruit Products** do You Eat?

**High fibre** fruits are eaten with the skin where practical. Includes fresh, dried or canned.

**Low fibre** fruit products include juices.

**1**	**2**	**3**	**4**
□	□	□	□
Almost never	1 serve a day	2–3 serves a day	3 or more serves a day

7. Vegetables. How Many Servings of Vegetables do You Eat? Include Each Serve of Vegetables, Counting a Tossed Salad as One Serve.

**1**	**2**	**3**	**4**
□	□	□	□
Almost never	1 serve a day	2–3 serves a day	3 or more serves a day

8. Fast Foods. How often do You Eat Foods such as Hamburgers, Pies, Chips, Fried Chicken, Hot Dogs, Sausage Rolls, Fried Asian Take-Aways?

**1**	**2**	**3**	**4**
□	□	□	□
Every day	Several times a week	Occasionally, not more than once every few weeks	Seldom or never

9. Salty Foods. How often do You Eat Soy Sauce, Crisps or Other Savoury Snacks, Salted Crackers, Salted Peanuts, Salt Cured Foods such as Bacon, Salami, Ham, *etc.*?

**1**	**2**	**3**	**4**
□	□	□	□
Never	Occasionally	Most of the time	Always

10. Salt. How often do You Add Salt to Your Food?

**1**	**2**	**3**	**4**
□	□	□	□
Always	Most of the time	Occasionally	Never

11. During the Past Month, How Many Days did You Drink Alcoholic Beverages?

**1**	**2**	**3**	**4**	**5**
□	□	□	□	□
0–5	6–10	11–15	16–20	>20

12. During the Past Month, How Many Times did You Have 5 or More Drinks per Occasion?

**1**	**2**	**3**	**4**	**5**
□	□	□	□	□
0–2	3–4	6–8	9–11	9–11

13. On the Average, How Many Glasses of Beer, Wine or Spirits, do You Consume a Week?

	**1**	**2**	**3**	**4**	**5**
	0–5	6–10	11–15	16–20	>20
**(a) Beer**	□	□	□	□	□
**(b) Wine**	□	□	□	□	□
**(c) Spirits**	□	□	□	□	□
